# Effect of Balint training in gastroenterology intern nurse practitioners: A systematic review protocol

**DOI:** 10.1097/MD.0000000000018129

**Published:** 2019-11-27

**Authors:** Wan-lu Liu, Zheng-ri Zhu, Chen Chen, Jing Li, Olga Moreno

**Affiliations:** a Department of Nursing Care; b Second Ward of Gastroenterology Department; c First Ward of Orthopedics Department, The Affiliated Hongqi Hospital of Mudanjiang Medical University, Mudanjiang, 157011, China; d School of Social and Community Medicine, University of Bristol, Bristol, BS81TH, UK.

**Keywords:** Balint training, effect, intern nurse, safety

## Abstract

**Background:**

This study aims to assess the effect of Balint training (BT) in gastroenterology intern nurse practitioners (GINP) systematically.

**Methods:**

This study will search EMBASE, MEDLINE, PsycINFO, Web of Science, Cochrane Library, Cumulative Index to Nursing and Allied Health Literature, Allied and Complementary Medicine Database, and China National Knowledge Infrastructure from inception to the September 30, 2019 with no language limitation. In addition, we will also search grey records, such as conference abstracts and dissertations. Study quality will be checked using Cochran risk of bias tool. Statistical analysis will be performed using RevMan 5.3 software.

**Results:**

This study will systematically evaluate the effect of BT in GINP and will provide evidence to judge whether BT is effective for GINP clinically.

**Conclusion:**

The results of this study may provide helpful evidence of BT in GINP in the clinical training.

## 1 Introduction

Balint training (BT) is a group-training method, which often consists of 6 to 12 physicians and 2 group facilitators.^[1–3]^ It refers to a method designed to helping physicians better understand their roles in the doctor–patient relationship, and also help to enhancing their interpersonal skills.^[4–6]^ It is also a patient-centered approach, which helps to aware and understand of how physicians’ emotions impact patients’ state of mind, rather than just deal with the diagnosis and treatment alone for the patients.^[7–11]^ Previous studies have reported that BT can help physicians to prevent their dimensions of emotional exhaustion, depersonalization, behavioral and psychological disorders, such as depression and anxiety.^[12–14]^ It can be applied for a variety of clinical practitioners training, including gastroenterology intern nurse practitioners (GINP).^[15–26]^ Although several studies have assessed the effect of BT for GINP, no results are consistent from those studies.^[18–20,23]^ Thus, this study will systematically explore the effect of BT for GINP.

## 2 Methods

### 2.1 Ethics and dissemination

This study will not use individual patient data, thus no ethics approval is required. This study will be published at a peer-reviewed journal or a conference presentation.

### 2.2 Eligibility criteria

#### 2.2.1 Study types

This study will include randomized controlled trials (RCTs) on assessing the effect of BT in GINP. Any other non-RCTs will be excluded.

#### 2.2.2 Intervention types

The participants in the experimental must undergo BT alone.

However, the participants in the control group can receive any interventions, except BT.

#### 2.2.3 Participant types

Participants of GINP who receive BT will be included without any limitations of their gender, age, and economic status.

#### 2.2.4 Outcome measurement

The primary outcomes are psychological disorders, including depression and anxiety, as measured by Hamilton Depression Scale or Hamilton Anxiety Scale, or any other relevant scales.

The secondary outcomes are clinical knowledge or skills, as measured using mini-Clinical Evaluation Exercise (mini-CEX) or any instruments; and satisfaction, as measured by any relevant surveys or scores; and examination scores.

### 2.3 Search strategy

We will comprehensively search for studies published in the following bibliographic databases: MEDLINE, EMBASE, PsycINFO, Web of Science, Cochrane Library, Cumulative Index to Nursing and Allied Health Literature, Allied and Complementary Medicine Database, and China National Knowledge Infrastructure, which cover records in the domains of health cares, medicine, nursing, allied health, health education and its training. Study searches through those databases will cover publication from their inception to the September 30, 2019 with no language limitation. The detailed search strategy for MEDLINE is presented in Table 1. We will also adapt similar search strategies to any other bibliographic electronic databases. In addition, we will also search grey records, such as conference abstracts, dissertations and reference lists of relevant reviews.

**Table 1:**
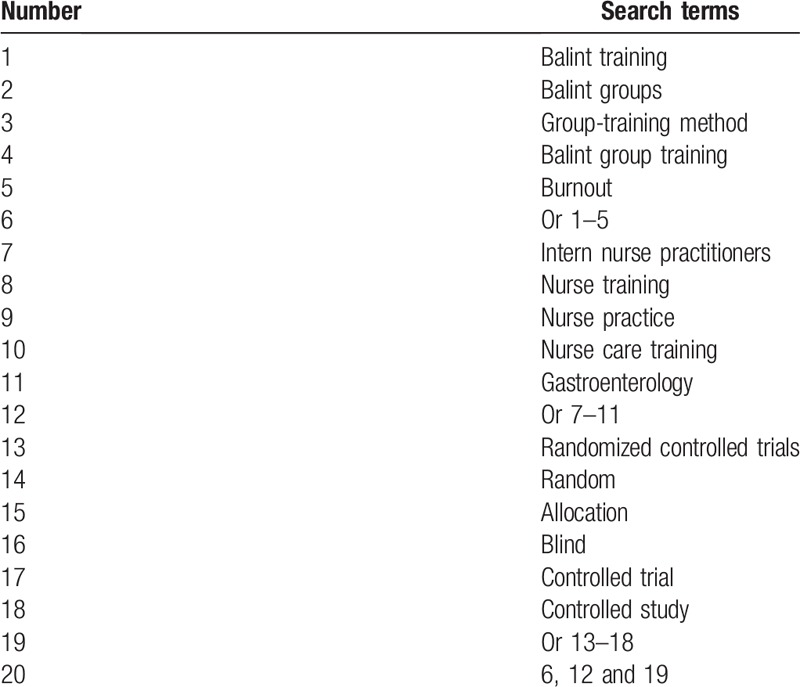
Search strategy for database of MEDLINE.

### 2.4 Study selection

Two authors will carry out study selection independently and respectively. First, titles and abstracts of all identified records will be scanned to remove any irrelevant and duplicated studies. Second, we will collect full-text of all remaining articles to further judge if they meet all inclusion criteria. All excluded studies will be recorded with specific reasons. We will demonstrate the whole process of study selection in the flowchart.

### 2.5 Data collection and management

Two authors will independently extract information from the included studies. Any differences and discrepancies will be reconciled with the help of a third author through discussion. The extracted information include first author, country of study, research study, research setting, participant characteristics, sample size and methods, research methods, interventions, controls, outcome measurements, and any other associated information.

### 2.6 Dealing with missing data

If any missing or unclear data will be identified, we will contact primary authors to inquire those data. If we cannot get reply, we will analyze available data and will discuss its potential effects.

### 2.7 Study quality

Two authors will independently evaluate research quality. Any discrepancies between two authors will be solved by consensus through arbitration by a third author. We will use Cochrane risk of bias tool to assess the study quality for each included study. We will assess each included study through 7 aspects, and each item will be assessed as “high,” “unclear,” or “low” risk of bias.

### 2.8 Data synthesis

We will use RevMan 5.3 software to perform statistical analysis. Continuous data will be calculated as standardized mean difference and 95% confidence intervals (CIs). Dichotomous data will be exerted as risk ratio and 95% CIs. We will calculate *I*^2^ statistic to determine the proportion of variation in effect size across included trials due to the heterogeneity regarding *I*^2^ ≤ 50% as acceptable heterogeneity, and *I*^2^ > 50% as obvious heterogeneity. If *I*^2^ ≤ 50%, we will use a fixed-effect model, and will conduct meta-analysis. If *I*^2^ > 50%, we will utilize a random-effect model, and will carry out subgroup analysis to investigate its possible reasons. If there is still obvious heterogeneity after subgroup analysis, we will report outcome results as a narrative summary.

### 2.9 Subgroup analysis

We will perform subgroup analysis according to the different interventions, comparators, and outcomes.

### 2.10 Sensitivity analysis

Sensitivity analysis will be conducted to check robustness of synthesized results by excluding studies with high risk of bias.

### 2.11 Reporting bias

If there are at lest 10 studies, we will conduct Funnel plot and Egger's regression test to identify any possible reporting bias.

## 3 Discussion

Previous studies have reported BT as a required component of the clinical practice. They hypothesized that BT has been used for the management of GINP.^[18–20,23]^ However, there are still not consistent results regarding this issue. Thus, considering several previous studies on investigating the effect of BT for GINP through measuring outcomes, such as mini-CEX, this study will systematically and comprehensively assess the effect of BT for GINP. The results of this study are expected to provide helpful evidence of BT for GINP.

## Author contributions

**Conceptualization:** Wan-lu Liu, Chen Chen, Jing Li, Olga Moreno.

**Data curation:** Wan-lu Liu, Zheng-ri Zhu, Chen Chen, Jing Li.

**Formal analysis:** Wan-lu Liu, Zheng-ri Zhu, Chen Chen.

**Funding acquisition:** Wan-lu Liu.

**Investigation:** Jing Li.

**Methodology:** Wan-lu Liu, Zheng-ri Zhu, Chen Chen, Olga Moreno.

**Project administration:** Jing Li.

**Resources:** Wan-lu Liu, Zheng-ri Zhu, Chen Chen.

**Software:** Wan-lu Liu, Zheng-ri Zhu.

**Supervision:** Jing Li.

**Validation:** Wan-lu Liu, Zheng-ri Zhu, Chen Chen, Olga Moreno.

**Visualization:** Wan-lu Liu, Chen Chen, Jing Li.

**Writing – original draft:** Wan-lu Liu, Zheng-ri Zhu, Jing Li.

**Writing – review & editing:** Wan-lu Liu, Zheng-ri Zhu, Chen Chen, Jing Li, Olga Moreno.

## References

[1] Omer S McCarthy G Reflective practice in psychiatric training: Balint groups. Ir J Psych Med 2010;27:115–6. 10.1017/S0790966700001269 30282198

[2] Adams KE O’Reilly M Romm J Effect of Balint training on resident professionalism. Am J Obstet Gynecol 2006;195:1431–7. 16996457 10.1016/j.ajog.2006.07.042

[3] Johnson AH Brock CD Hueston WH Resident physicians who continue Balint training: a longitudinal study 1982–1999, part II. Fam Med 2004;36:234–5. 15057607

[4] Johnson AH Brock CD Hueston WJ Resident physicians who continue Balint training: a longitudinal study 1982–1999. Fam Med 2003;35:428–33. 12817871

[5] Shanafelt TD Bradley KA Wipf JE Burnout and self-reported patient care in an internal medicine residency program. Ann Intern Med 2002;136:358–67. 11874308 10.7326/0003-4819-136-5-200203050-00008

[6] Cataldo KP Peeden K Geesey ME Association between Balint training and physician empathy and work satisfaction. Fam Med 2005;37:328–31. 15883898

[7] Turner AL Malm RL A preliminary investigation of Balint and non-Balint behavioral medicine training. Fam Med 2004;36:115–7. 14872358

[8] Bar-Sela G Lulav-Grinwald D Mitnik I “Balint group” meetings for oncology residents as a tool to improve therapeutic communication skills and reduce burnout level. J Cancer Educ 2012;27:786–9. 22923383 10.1007/s13187-012-0407-3

[9] Romani M Ashkar K Burnout among physicians. Libyan J Med 2014;9:23556. 10.3402/ljm.v9.23556 PMC3929077 24560380

[10] Kjeldmand D Holmström I Rosenqvist U Balint training makes GPs thrive better in their job. Patient Educ Couns 2004;55:230–6. 15530759 10.1016/j.pec.2003.09.009

[11] Bellet P Maloney M The importance of empathy as an interviewing skill in medicine. JAMA 1991;266:1831–2. 1909761

[12] Ghetti C Chang J Gosman G Burnout, psychological skills, and empathy: Balint training in obstetrics and gynecology residents. J Grad Med Educ 2009;1:231–5. 21975984 10.4300/JGME-D-09-00049.1 PMC2931236

[13] Samuel OW Aims and objectives of Balint training. J Balint Soc 1987;15:23–5.

[14] Benson J Magraith K Compassion, fatigue and burnout. Aust Fam Physician 2005;34:497–8. 15931410

[15] Zou QY Liu MQ Meng LM Application of Balint group training activities in clinical nurses’ pressure release. Chin Contemporary Med 2019;26:196–9.

[16] Ye LL Xu YX Yao Q Applying the training model of Balint Group to improve nurses’ self-efficacy. Psychol Monthly 2019;14:235–6.

[17] Wang QY Shao LP Wang JX The effect of group psychological intervention mode on nurses’ self-efficacy and emotional labor. J Xinjiang Med Univ 2019;42:266–8.

[18] Li D The impact of the Balint Group training model on communication skills and emotional intelligence scores of new nurses in outpatient clinics. Chin J Med Sci 2019;9:143–6.

[19] Zhang XY Ye XY Zhou HJ Analysis of the intervention effect of the training service of the Balint Group on the position and communication ability of new nurses. Chin Remedies Clinics 2019;19:313–5.

[20] Cheng D Guo W The influence of the training method of the Balint Group on the empathy of the new nurses. Nurs Res 2019;33:160–2.

[21] Sun HM Guo L The application effect of the Balint group model to improve the clinical communication ability of outpatient nurses. Tianjin Nurs 2018;26:211–3.

[22] Zhu XM Yao J Xu LT Study on the role of Balint Group training in improving nurses’ emotional intelligence and communication skills. World Med Information Digest 2017;17:10–1.

[23] Chen Y The impact of the Balint Group training model on the communication ability of nurses and nurses in new nurses. Shenzhen J Integr Tradit Chin Western Med 2017;27:195–6.

[24] Dong JW Sha LY Yi J The role of Balint group training in enhancing nurses’ emotional intelligence and communication ability. J Nurs Sci 2016;31:73–5.

[25] Zhai YJ Wang YB Huang YT Observation of the application effect of the Balint Group in relieving the pressure of nurses in quality nursing wards. Qilu Nurs J 2016;22:116–7.

[26] Pang JY Lu L Chen CH The impact of the Balint Group and related training on communication skills and coping styles of medical staff. Chin J Nurs 2015;32:60–3.

